# Antigenic and genetic characterization of a divergent African virus, Ikoma lyssavirus

**DOI:** 10.1099/vir.0.061952-0

**Published:** 2014-05

**Authors:** Daniel L. Horton, Ashley C. Banyard, Denise A. Marston, Emma Wise, David Selden, Alejandro Nunez, Daniel Hicks, Tiziana Lembo, Sarah Cleaveland, Alison J. Peel, Ivan V. Kuzmin, Charles E. Rupprecht, Anthony R. Fooks

**Affiliations:** 1 Animal Health and Veterinary Laboratories Agency (AHVLA), Weybridge, UK; 2 Institute of Biodiversity, Animal Health and Comparative Medicine, University of Glasgow, Glasgow, UK; 3 Disease Dynamics Unit, Department of Veterinary Medicine, University of Cambridge, UK; 4 Global Alliance for Rabies Control, Manhattan, KS, USA; 5 Ross University School of Veterinary Medicine, St Kitts; 6 Department of Clinical Infection, Microbiology and Immunology, University of Liverpool, Liverpool, UK

## Abstract

In 2009, a novel lyssavirus (subsequently named Ikoma lyssavirus, IKOV) was detected in the brain of an African civet (*Civettictis civetta*) with clinical rabies in the Serengeti National Park of Tanzania. The degree of nucleotide divergence between the genome of IKOV and those of other lyssaviruses predicted antigenic distinction from, and lack of protection provided by, available rabies vaccines. In addition, the index case was considered likely to be an incidental spillover event, and therefore the true reservoir of IKOV remained to be identified. The advent of sensitive molecular techniques has led to a rapid increase in the discovery of novel viruses. Detecting viral sequence alone, however, only allows for prediction of phenotypic characteristics and not their measurement. In the present study we describe the *in vitro* and *in vivo* characterization of IKOV, demonstrating that it is (1) pathogenic by peripheral inoculation in an animal model, (2) antigenically distinct from current rabies vaccine strains and (3) poorly neutralized by sera from humans and animals immunized against rabies. In a laboratory mouse model, no protection was elicited by a licensed rabies vaccine. We also investigated the role of bats as reservoirs of IKOV. We found no evidence for infection among 483 individuals of at least 13 bat species sampled across sites in the Serengeti and Southern Kenya.

## Introduction

The discovery of novel viruses has flourished with the advent of highly sensitive molecular detection techniques (Bexfield & Kellam, 2011; Lipkin & Firth, 2013). These include metagenomic studies of the viral flora of healthy animals aimed at predicting transmission risks to other species, and the detection of pathogens in clinical samples and excreta for diagnosis (Bodewes *et al.*, 2013; Ge *et al.*, 2012; Li *et al.*, 2011; Phan *et al.*, 2011; van den Brand *et al.*, 2012). Such studies provide valuable information on the presence and variability of viral pathogens in different animal populations. However, the detection of viral nucleic acid alone provides limited information on the zoonotic and pathogenic potential of such viruses.

Recent expansion of the genus *Lyssavirus*, within the family *Rhabdoviridae*, provides an example of rapid increase in the number of novel viruses identified, with the number of lyssavirus species doubling in the past 10 years. There are 12 lyssavirus species classified by the International Committee on Taxonomy of Viruses, with two awaiting classification (Dietzgen *et al.*, 2011) and genetic evidence for a further putative lyssavirus detected in Spain (Aréchiga Ceballos *et al.*, 2013). Lyssaviruses are divided into at least two phylogroups, based on genetic and antigenic distance (Badrane *et al.*, 2001; Evans *et al.*, 2012; Hanlon *et al.*, 2005; Horton *et al.*, 2010; Table S1, available in the online Supplementary Material). All lyssaviruses cause rabies, which remains untreatable once clinical signs develop (Johnson *et al.*, 2010) with a near 100 % fatality rate. Therefore, prevention of disease relies upon pre- and post-exposure prophylaxis through administration of vaccine and immune globulin. All licensed vaccines and immune globulin treatments are based on inactivated preparations or antibodies directed against ‘classical’ rabies virus (RABV). These preparations elicit protective immunological responses to all RABV variants tested, but have variable efficacy against several other lyssaviruses (Badrane *et al.*, 2001; Brookes *et al.*, 2005a; Hanlon *et al.*, 2005; Lodmell *et al.*, 1995). Although difficult to quantify, this variation is related to antigenic distances of such lyssaviruses from vaccine strains (Badrane *et al.*, 2001; Evans *et al.*, 2012; Hanlon *et al.*, 2005; Horton *et al.*, 2010). A strong immunological response to standard rabies vaccine has been shown to protect against lyssaviruses that are classified within phylogroup I, but not against viruses classified within other phylogroups (Fooks, 2004).

In 2009, an African civet (*Civettictis civetta*), with clinical signs of rabies, was killed in the Serengeti National Park (SNP), Tanzania, after biting a child. The child received appropriate wound care and post-exposure rabies prophylaxis and remained healthy. Direct antigen-detection tests on brain samples from the civet confirmed infection with a lyssavirus, and genetic analysis demonstrated that the causative agent (Ikoma lyssavirus, IKOV) was highly divergent from all lyssaviruses characterized previously (Marston *et al.*, 2012a, b). This finding was particularly significant as dogs are not permitted in the SNP, and the area of the park where the civet was encountered had been free from canine rabies for over 8 years (Lembo *et al.*, 2008). Prediction of antigenic distance and by extrapolation, degree of protection, provided by rabies vaccine strains using sequence data alone is not precise, but the degree of genetic divergence suggested that currently available vaccines would not be likely to be able to confer protection against IKOV (Evans *et al.*, 2012; Horton *et al.*, 2010).

The African civet, a solitary scavenger, does not fill the ecological niche nor have the population dynamics typical of a carnivore rabies host (Cleaveland & Dye, 1995; Estes, 1992). The few published reports of rabies in civets have been either dog- or mongoose-associated RABV variants, suggestive of spillover rather than maintenance of infection (Sabeta *et al.*, 2008). Despite dogs being responsible for the majority of human rabies cases worldwide, a variety of RABV lineages and numerous diverse lyssavirus species have been detected in wildlife, and particularly in a range of bat species (Banyard *et al.*, 2011; Hayman *et al.*, 2012; Kuzmin *et al.*, 2010). Antibodies to a phylogenetically related lyssavirus, West Caucasian bat virus (WCBV), have been detected in *Miniopterus* spp. bats in Kenya close to the border with Tanzania (Kuzmin *et al.*, 2008a), but there are no published studies showing evidence for lyssavirus antibodies in bats in the SNP. Therefore, with evidence for bat reservoirs for closely related viruses, it is possible that the natural host and reservoir for IKOV is a bat species.

The detection of genetic material from a new virus causing rabies in a potential spillover host, with no known reservoir, requires further investigation. Here we describe the isolation and characterization of viable IKOV *in vivo* and *in vitro*, assess the degree of serological cross-neutralization of IKOV with RABV and WCBV, and assess the efficacy of rabies vaccination against IKOV in an animal model. We also present results of surveillance for IKOV in bats living in close proximity to the location of the index case.

## Results

### *In vitro* characterization

IKOV was not easily isolated from clinical material. Repeated blind passage of civet brain homogenate in neuroblastoma cell culture failed to amplify viable virus. IKOV was initially isolated through intracranial (IC) inoculation of 4-week-old CD-1 mice and stocks of virus were then generated by six serial passages in murine fibroblast (baby hamster kidney, BHK-21) cells. The fluorescent antibody test (FAT) was performed on brain smears post-mortem. Viral antigens were detected using FITC-conjugated anti-rabies nucleocapsid protein antibodies. This preparation of antibodies has demonstrated high sensitivity and specificity in detecting lyssaviruses (Robardet *et al.*, 2012). Virus titres at passage five (10^4.42^, TCID_50_ ml^−1^) and passage six (10^4.8^ TCID_50_ ml^−1^) were comparable to those seen with other lyssaviruses (Brookes *et al.*, 2005a; Horton *et al.*, 2010; Koraka *et al.*, 2012).

A modified fluorescent antibody virus neutralization (mFAVN) test was used to assess the degree of cross-neutralization between IKOV and other lyssaviruses (Brookes *et al.*, 2005b; Cliquet *et al.*, 1998). Sera from four humans and ten dogs vaccinated with commercial rabies vaccines demonstrated negligible levels of cross-neutralization against IKOV (Table 1). Three of these individuals had exceptionally high reciprocal antibody titres against RABV (Challenge Virus Standard, CVS) of over 1 : 30 000, and yet had reciprocal titres of less than 1 : 8 against IKOV, indistinguishable from negative controls and therefore effectively negative. In addition, neat human rabies immune globulin (HRIG), at a concentration of 270 IU ml^−1^, showed no detectable neutralization of IKOV. Sera from mice inoculated with IKOV that showed reciprocal antibody titres of 1 : 27 to 1 : 420 against IKOV failed to neutralize CVS in standard FAVN tests. Furthermore, rabbit serum with a neutralizing antibody titre of over 1 : 1000 against WCBV failed to neutralize IKOV (Table 1). This lack of detectable cross-neutralization precluded accurate positioning of IKOV on an antigenic map developed previously (Horton *et al.*, 2010).

**Table 1.** Cross-neutralization of sera from rabies vaccinated humans and animals against IKOV Neutralizing antibody titres against RABV (Challenge virus standard, CVS) and IKOV in reciprocal 50 % end point titres. International units (IU) are given for rabies by comparison to a standard control (not applicable to IKOV). Mice 3, 4 and 5 were inoculated IM with IKOV at 10^3.8^ TCID_50_ ml^−1^ and survived. Rabbit 821 is rabbit anti-WCBV serum with a reciprocal titre of 1448 against WCBV. nd, not done.

	Virus		
	CVS (100 TCID_50_)		IKOV (100 TCID_50_)
Sample ID	IU ml^−1^	Reciprocal titre	Reciprocal titre
HUMAN - 1	23	959	<8
HUMAN - 2	31	1 263	<8
HUMAN - 3	1 093	34 092	<8
HUMAN - 4	1 093	34 092	<8
DOG 1	53	1 263	<8
DOG 2	41	960	<8
DOG 3	1 094	25 904	<8
DOG 4	122	2 878	<8
DOG 5	365	8 635	<8
DOG 6	1	16	<8
DOG 7	1 094	34 109	<8
DOG 8	364	11 364	<8
DOG 9	14	421	<8
DOG 10	41	1 263	<8
HRIG	270	nd	<16
Mouse 3	<0.2	<16	27
Mouse 4	<0.2	<16	420
Mouse 5	nd	nd	81
Rabbit 821	<0.2	<16	<8

### *In vivo* pathogenesis

**Fig. 1.**
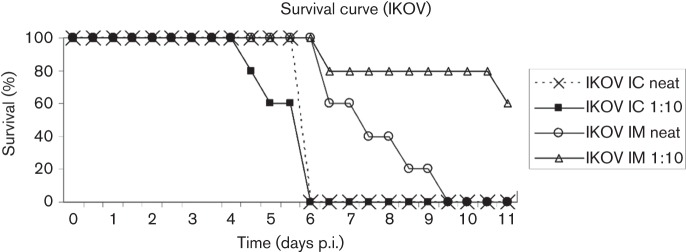
Survival chart showing percentage survival in days post-inoculation (p.i) for groups of five mice inoculated either intracranially (IC) or intramuscularly (IM) with neat IKOV (10^4.8^ TCID_50_ ml^−1^) or a 1/10 dilution (10^3.8^ TCID_50_ ml^−1^). Mice not clinically affected after 11 days were still healthy when euthanized at 28 days and negative for lyssavirus antigens post-mortem.

All mice inoculated IC with 0.03 ml tissue culture passaged IKOV at high dose (10^4.8^ TCID_50_ ml^−1^) or a 10-fold dilution (10 ^3.8^ TCID_50_ ml^−1^) succumbed to challenge with an incubation period of between 4.5 and 6 days, with no apparent dose effect. There was, however, a detectable dose effect (albeit not statistically significant at the 95 % level) for the intramuscularly (IM) challenged mice, with 5/5 of the mice challenged IM with 0.03 ml IKOV at 10^4.8^ TCID_50_ ml^−1^ succumbing between 6.5 and 9 days, but only 2/5 of the group receiving IKOV at 10 ^3.8^ TCID_50_ ml^−1^ succumbing, with incubation periods of 7 and 11 days (Fischer’s exact test, *P* = 0.08; Fig. 1). The remaining three mice inoculated IM with the lower dose had no detectable lyssavirus antigens in the brain post-mortem, but had seroconverted when subjected to euthanasia at 28 days (Table 1). Prodromal clinical signs were similar to those recorded for other lyssaviruses in mice, including reduced appetite, ruffled fur and hunched posture (Healy *et al.*, 2013). Mice then progressed to either hind limb paralysis, or hyperexcitability and convulsions, with a predominance of the latter, and were subjected to euthanasia.

### Pathology

**Fig. 2.**
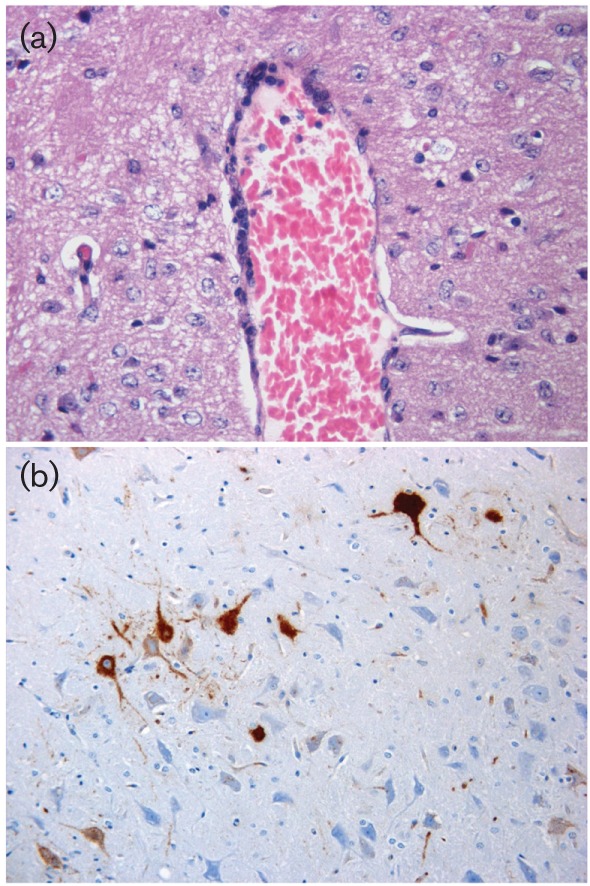
Histopathological examination of brain from IKOV-infected mice. (a) Perivascular cuffing in the cortex composed of few inflammatory cells. Inflammatory changes were minimal to mild and perivascular cuffs rare and not prominent. Image shows haematoxylin and eosin staining at 400× magnification. (b). IHC demonstration of viral antigens in the medulla (brown labelling). Image shows IHC at 400× magnification.

Mice inoculated with IKOV and subject to euthanasia at a clinical score of 2 to 3 had developed non-suppurative encephalitis (Fig. 2a). The inflammatory changes were very mild, with occasional perivascular cuffing but without notable gliosis or degenerative changes in neurons. IKOV antigens were observed using immunohistochemistry (IHC) in the perikaryon and neuropil in all brain regions examined, with the majority of staining in the medulla and only rare and dispersed antigens in the cortex and thalamus (Fig. 2b).

### Vaccine challenge experiments

Nineteen mice were vaccinated with one dose of a commercial rabies vaccine, and 18 of these had seroconverted against RABV by day 21 (Titre range 0.70–256 IU ml^−1^), confirming an adequate response to vaccination. Mice vaccinated under identical conditions were previously demonstrated to be protected against IC challenge with RABV (Brookes *et al.*, 2005a). All 19 mice challenged IC with IKOV in a modified National Institutes of Health (NIH) test developed rabies, as did all the unvaccinated controls. The presence of IKOV antigens and RNA was confirmed in the brains of challenged mice.

### Genetic characterization

**Fig. 3.**
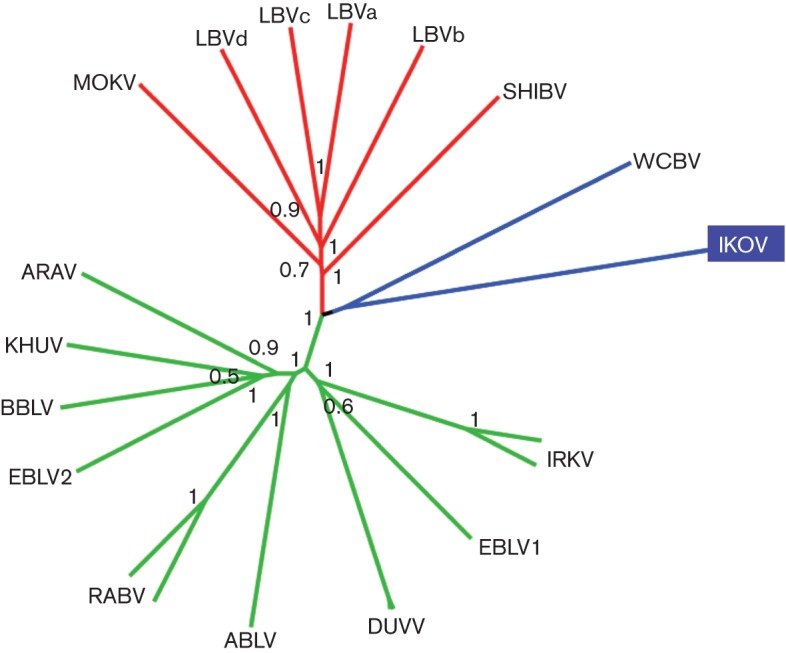
Neighbour-joining phylogenetic tree of concatenated coding areas of five genes (N+P+M+G+L) of IKOV compared to representative lyssaviruses from all other species (Table S1). Branches are coloured by phylogroup: green, phylogroup one; red, phylogroup two; and blue, uncharacterized. Boostrap values (proportion of 1000 replicates) are given at each node.

Comparison of the full genome of IKOV with the full genome of representatives of other lyssaviruses allows interpretation of genetic relationships among the lyssaviruses (Fig. 3). Analysis of the full genomes supports the previously reported relationship of WCBV and IKOV. Lagos bat virus (LBV), Shimoni Bat virus (SHIBV) and Mokola virus (MOKV), all previously characterized as phylogroup II, comprise a strongly supported separate monophyletic group. The European bat lyssaviruses have separate ancestors. European bat lyssavirus type-1 (EBLV-1) shares a common ancestor with Duvenhage virus (DUVV) and Irkut virus (IRKV), separate from the ancestor common to European bat lyssavirus type-2 (EBLV-2) and the remaining lyssavirus species [RABV, Aravan virus (ARAV), Khujand virus (KHUV), Bokeloh bat lyssavirus (BBLV) and Australian bat lyssavirus (ABLV)]. IKOV and WCBV form a monophyletic group outside of the phylogroup I and phylogroup II lyssaviruses with significant bootstrap support, although they are separated by long genetic distance (63.4 % nucleotide identity for the concatenated coding gene sequences).

### Sampling of bats in Africa for antibodies to IKOV

The common and ubiquitous free-tailed bats (*Chaerephon pumilus*) in the immediate vicinity of the index IKOV case in the SNP were roosting in the roof space of the few human dwellings, used by park employees and research scientists. These were therefore the priority for sampling in Tanzania, and were the only species encountered during sampling in SNP in 2012. Sera were available from 25 of these free-tailed bats (*C. pumilus*) sampled within SNP, and in two settlements in close proximity to the north-western boundary of the SNP. Two other species (*Hipposideros* sp., *n* = 16, and *Nycteris* sp., *n* = 1) were also sampled in these settlements. In Kenya, 441 individuals from at least 10 different bat species were caught in multiple locations during 2011, in the framework of the Global Disease Detection Program of the US Centers for Disease Control and Prevention (CDC) (Table S2). These included 229 *Miniopterus* spp., of which 48 neutralized WCBV (titre range 1.3–3.1 log_10_ ED_50_). All 483 of these sera from Tanzania and Kenya failed to show any neutralizing activity against IKOV (see Table S2 and Methods provided in the online Supplementary Material).

## Discussion

The discovery of a novel lyssavirus (IKOV) causing rabies in an African civet, in a wildlife-rich area with potential for wildlife–human interaction, required further investigation to assess its public and animal health significance. Here, we have demonstrated that peripheral pathogenicity of IKOV is comparable to that of RABV in a rodent model. The virus is antigenically distinct to all other lyssaviruses, and vaccination with rabies vaccine produced no cross-neutralizing antibodies in humans or animals, and did not elicit protection in an animal challenge model.

The G protein is the immunodominant lyssavirus protein, and comparison of the G coding sequence of IKOV with other lyssavirus species demonstrates 46–50 % and 52–55 % similarity at the nucleotide and amino acid levels, respectively (Evans *et al.*, 2012). Previous quantitative assessments of the effect of these genetic differences on antigenicity suggested that 72 % amino acid identity along the G ectodomain is a threshold for efficient cross-neutralization (Badrane *et al.*, 2001), and that a 4.8 % difference in amino acid sequence identity over the ectodomain region would cause, on average, a twofold difference in antibody titre against a virus (Horton *et al.*, 2010). Therefore, with only 47 % amino acid identity with CVS, we would expect to see no cross-neutralization between RABV and IKOV. Here we were able to confirm this hypothesis, demonstrating a complete lack of cross-neutralization in sera of humans and animals vaccinated from rabies, even if they demonstrated very high neutralizing titres against CVS. Similar studies of phylogroup I lyssaviruses showed reduced but significant neutralization of EBLVs relative to CVS (Brookes *et al.*, 2006; Malerczyk *et al.*, 2009), and even studies of the most divergent lyssavirus prior to this discovery, WCBV, showed a limited neutralization by sera of rabbits that had high neutralizing titres against CVS (Hanlon *et al.*, 2005; Horton *et al.*, 2010). In this study, we have demonstrated a lack of cross-neutralization between IKOV and WCBV, despite the viruses having a monophyletic relationship in phylogenetic reconstructions. Our observations suggest that demarcation of lyssaviruses into phylogroups may be more complex than a comparison of glycoprotein identity values and amount of cross-neutralization as proposed initially (Badrane *et al.*, 2001).

Crucially, HRIG at 270 IU ml^−1^ failed to show significant neutralization of IKOV (Table 1). In all category three exposures, HRIG is recommended as defined by the WHO (Anon, 2009), but these data demonstrated it is unlikely that HRIG would be effective against IKOV. This lack of protection afforded by rabies biologics reinforces the value of thorough wound cleaning and antiseptic treatment in cases of exposure to carnivores and bats (Anon, 2009).

The current gold standard test for assessment of rabies vaccine potency is the NIH test, which uses an IC challenge in mice vaccinated with serial dilutions of vaccine (Wilbur & Aubert, 1996). The test has been successfully modified for the assessment of protection against other viruses by using a single vaccine dilution, and mice vaccinated using this technique have previously been protected against IC challenge with RABV (Brookes *et al.*, 2005a; Wilbur & Aubert, 1996). However, all 19 mice vaccinated with commercial rabies vaccine and challenged with IKOV succumbed to disease, demonstrating that no protection was conferred. These results confirm the predictions obtained from the genetic and *in vitro* antigenic studies that rabies vaccines do not elicit protection against IKOV in mice and are therefore unlikely to provide protection in other mammals, including humans.

Analysis of full concatenated coding gene sequences of representative lyssaviruses showed similar topology to previous analyses based on partial N-gene sequences and glycoprotein sequences, suggesting that IKOV and WCBV form a monophyletic group, albeit with deeply rooted divergence (Evans *et al.*, 2012; Marston *et al.*, 2012a). One difference seen here is that in the previous partial N-gene analyses, MOKV, WCBV and IKOV formed a monophyletic group separate from LBV, which is not the case in this full genome comparison.

Knowledge of the reservoir of zoonotic diseases is important, to inform prevention of spillover infections. IKOV was detected in an African civet, an unlikely reservoir for a lyssavirus. Civets are mostly solitary scavengers, with presumed low contact rates (Estes, 1992). Consistent with this ecology is the detection of varied canid and mongoose variants of RABV in the few reported cases of rabies in civets, suggesting that civets are spillover hosts (Sabeta *et al.*, 2008). No further cases caused by IKOV have been detected, despite high levels of tourist and research-related activity in the area that have been sufficient to detect RABV in another wild carnivore since the index IKOV case. This suggests that the civet case did not result in a sustained chain of transmission. Nonetheless, a civet reservoir cannot be ruled out definitively, so vigilance for rabies in civets and other wild carnivore species still needs to be maintained, including virus characterization of any positive cases.

In light of the association of lyssaviruses with bats (Banyard *et al.*, 2011), attention has inevitably focused on bat populations in and around SNP to find the reservoir of IKOV. The IKOV-positive civet was encountered on the edge of the Serengeti plains, with few suitable roosting places for bats. Previous studies demonstrated up to 70 % seroprevalence of bats to various lyssaviruses (Harris *et al.*, 2009; Hayman *et al.*, 2012; Kuzmin *et al.*, 2010, 2008a, b; Turmelle *et al.*, 2009) but all bat sera tested in this study were negative for antibodies to IKOV. The limited sample size, species coverage and narrow time frame preclude robustly ruling out IKOV infection in bats. However, based on these data, and seroprevalence levels demonstrated in reservoir bat populations to other lyssaviruses, it is unlikely that IKOV was circulating in the local free-tailed bat population at the time of sampling. However, the presence of other bat species, the variable success of bat capture techniques for different species, and the possibility of long-distance bat movements, suggests that the reservoir of IKOV is still likely to be in a bat species. The most recently detected putative lyssavirus species, Lleida bat lyssavirus, was detected in a *Miniopterus schreibersii* bat in Spain, and is phylogenetically related to WCBV and IKOV (Aréchiga Ceballos *et al.*, 2013). *Miniopterus* spp. bats are also a putative reservoir for WCBV in Kenya (Kuzmin *et al.*, 2008a). There was a notable absence of *Miniopterus* spp. captured in the SNP, despite the species being detected in large numbers in neighbouring Kenya. All *Miniopterus* spp. bat sera from Kenya were negative for IKOV antibodies, but there are at least 12 species of *Miniopterus* bats described in Africa (IUCN, 2013), and some species roost mainly in caves and can travel long distances to feed. Sampling of cave-dwelling bats in and around the SNP, together with serological screening of other wildlife samples from the SNP, would be a logical next step to investigating potential reservoirs.

In the experimental animal model used in this study, the incubation period, clinical signs and pathology of IKOV infection were consistent with those caused by other lyssaviruses (Healy *et al.*, 2013), and these data therefore confirm that IKOV causes rabies. The peripheral pathogenicity of IKOV concurs with recent studies showing that substitution at a key domain of the glycoprotein (K/R333D) which is shared by IKOV and phylogroup II lyssaviruses, does not result in lower peripheral pathogenicity (Badrane *et al.*, 2001; Kgaladi *et al.*, 2013; Kuzmin *et al.*, 2010, 2008b; Markotter *et al.*, 2008), in contrast to initial data based on fewer isolates (Badrane *et al.*, 2001). These results are corroborated by the fact that IKOV caused clinical rabies in the civet from which it was originally isolated, and therefore has potential to cause encephalitis in other hosts. This raises the question as to why spillover events like this do not happen more frequently. One obvious possibility is that they do, but remain undetected. IKOV was identified during dedicated studies on rabies dynamics in Tanzania, but virus characterization is not routine in most regions of Africa, with positive cases assumed to be RABV. Alternatively, species constraints might limit the number of effective spillover infections with IKOV as was suggested for other bat lyssaviruses (Banyard *et al.*, 2011). Continued enhanced surveillance, including laboratory-based confirmation of diagnosis and virus characterization, is necessary to assess and mitigate the public and animal health threats posed by IKOV and other emerging viruses (Banyard *et al.*, 2013).

## Methods

### 

#### *In vitro*.

Civet brain material was stored frozen at the Tanzania National Parks veterinary field laboratory in the Serengeti from 2009 until 2011 and then shipped to AHVLA for virus isolation and characterization. Brain homogenates from the first mouse passage were used to inoculate BHK-21 cell cultures, and the virus was passaged to a high titre for *in vivo* analysis during six passages: 0.5 ml clarified 10 % mouse brain homogenate in PBS was added to 2 ml of a BHK-21 cell suspension at 2×10^5^ cells ml^−1^, and incubated at 37 °C for 20 min with intermittent agitation. The infected cells were then added to a cell culture flask with fresh Glasgow minimal essential medium supplemented with 10 % FBS and 10 % tryptose phosphate broth. Every 3–5 days the cells were disrupted using antibiotic–trypsin–versene and transferred to a new flask with fresh media, and uninfected cells at a 1 : 1 ratio of infected to uninfected cells. Virus titre was assessed at passages five and six using techniques described previously (Aubert, 1996) and calculated with the Spearman–Kärber method. Viral antigens were visualized in acetone-fixed cells by the direct fluorescent antibody test (FAT) using standard techniques (Dean *et al.*, 1996) with an FITC-conjugated antibody (FITC anti-rabies monoclonal globulin; Fujirebio Diagnostics).

A modified fluorescent antibody neutralization test (mFAVN) was developed and optimized for IKOV using tissue culture passaged virus supernatant (TCSN) (Horton *et al.*, 2010). A standard quantity of virus (100 TCID_50_ per 50 µl) was added to serial twofold dilutions of serum in duplicate, with the quantity of virus checked by back-titration on each test. Sera and virus were incubated with BHK-21 cells for 48 h before fixing in acetone and staining with FITC-conjugated antibody (Fujirebio Diagnostics). The 50 % end point serum dilution was calculated with the Spearman–Kärber method (Aubert, 1996). Mouse serum from the second mouse passage of IKOV was used as a positive control, and serum from uninfected mice was used as a negative control. A panel of sera from animals and human vaccinees, with proven high serum neutralizing antibody levels to RABV, were tested for their ability to neutralize IKOV (Table 1). A rabbit anti-WCBV serum (rabbit 821), which neutralized WCBV at a reciprocal titre of 1 : 1448, was also tested in the same assay (Horton *et al.*, 2010).

#### *In vivo*.

All experimental work in animal models was undertaken under Home Office Licence after independent ethical review. Virus was first isolated from clinical material in 4-week-old OF1 mice, after unsuccessful isolation attempts in cell culture despite repeated passages using the rabies tissue culture inoculation test (RTCIT; Marston *et al.*, 2012a). Clarified civet brain homogenate (30 µl) was inoculated into mice (*n* = 5) IC. Mice were monitored twice daily using a clinical scoring system from 1 to 5 (Healy *et al.*, 2013) and subjected to euthanasia as soon as they progressed beyond clinical score 1. Brains of mice were tested by FAT. To investigate the virulence of IKOV, four groups of five OF1 mice were challenged with either neat TCSN (high dose, neat 10^4.8^ TCID_50_ ml^−1^) or a 10-fold dilution (low dose 10^3.8^ TCID_50_ ml^−1^) by either IC or IM inoculation, followed by twice daily observation and euthanasia when clinical score progressed beyond 1. IHC demonstration of RABV antigens in post-mortem samples from brains of mice was performed as described previously (Hicks *et al.*, 2009).

To assess protection provided by vaccination, a group of 19, 4-week-old OF1 mice were vaccinated with 0.5 ml of a commercially available vaccine (VERORAB, Sanofi Pasteur MSD) by intraperitoneal injection, as standard. A group of five control mice were left unvaccinated. After 21 days a blood sample was taken from the tail vein of each mouse and the level of RABV neutralizing antibodies was assessed using a CVS pseudotype assay (Wright *et al.*, 2009). The mice were then challenged IC with IKOV TCSN at 10^4.8^ TCID_50_ ml^−1^, observed twice daily and subjected to euthanasia at the first sign of disease.

#### Molecular analyses.

Nucleic acids were extracted using Trizol (Invitrogen) according to the manufacturer’s instructions. A pan-lyssavirus real-time PCR assay using iScript (Bio-Rad) was then used to test for the presence of lyssavirus RNA in mouse brain (Hayman *et al.*, 2011).

#### Phylogenetic analyses.

The full genome of IKOV was previously determined directly from clinical civet brain material using a combination of next generation sequencing and Sanger sequencing methods (GenBank accession number JX193798; Marston *et al.*, 2012b). In this report, concatenated gene sequences for all genes (N+P+M+G+L) from representatives from all lyssavirus species were compared to those of IKOV using neighbour-joining analysis, thereby avoiding the potential issue of different evolutionary rates between genes. Complete concatenated sequences were aligned using the clustal w algorithm in mega version 4, a neighbour-joining tree was constructed using p-distances with 1000 boostrap replicates, and visualized using FigTree(v1.2).

#### Field sampling of bats in Africa.

To investigate the possibility of a local bat reservoir for IKOV, after ethical review and under permit from local authorities, bats were sampled both in the immediate vicinity of the index IKOV case, and in areas of human habitation (Bunda town and Fort Ikoma village) close to the north-western border of the SNP in Tanzania (*n* = 42) in July 2012. Sera were also analysed from bats sampled in multiple locations in Southern Kenya (*n* = 441) as part of a separate study (Table S2). All 483 serum samples from at least 11 bat species were tested for the presence of neutralizing antibodies to IKOV using mFAVN and modified rabies fluorescent focus inhibition test (see Methods section in the online Supplementary Material).

## References

[r1] Anon **(**2009**).** Current WHO Guide for Rabies Pre and Post-exposure Prophylaxis in Humans. Geneva: World Health Organisation

[r2] Aréchiga Ceballos N. Vázquez Morón S. Berciano J. M. Nicolás O. Aznar López C. Juste J. Rodríguez Nevado C. Aguilar Setién A. Echevarría J. E. **(**2013**).** Novel lyssavirus in bat, Spain. Emerg Infect Dis 19, 793–795 10.3201/eid1905.121071 23648051 PMC3647500

[r3] Aubert M. **(**1996**).** Methods for the calculation of titres. In Laboratory Techniques in Rabies, pp. 445–459 Edited by Meslin F. X. Kaplan M. M. Koprowski H. Geneva: World Health Organisation

[r4] Badrane H. Bahloul C. Perrin P. Tordo N. **(**2001**).** Evidence of two Lyssavirus phylogroups with distinct pathogenicity and immunogenicity. J Virol 75, 3268–3276 10.1128/JVI.75.7.3268-3276.2001 11238853 PMC114120

[r5] Banyard A. C. Hayman D. Johnson N. McElhinney L. Fooks A. R. **(**2011**).** Bats and lyssaviruses. Adv Virus Res 79, 239–289 10.1016/B978-0-12-387040-7.00012-3 21601050

[r6] Banyard A. C. Horton D. L. Freuling C. Müller T. Fooks A. R. **(**2013**).** Control and prevention of canine rabies: the need for building laboratory-based surveillance capacity. Antiviral Res 98, 357–364 10.1016/j.antiviral.2013.04.004 23603498

[r7] Bexfield N. Kellam P. **(**2011**).** Metagenomics and the molecular identification of novel viruses. Vet J 190, 191–198 10.1016/j.tvjl.2010.10.014 21111643 PMC7110547

[r8] Bodewes R. van der Giessen J. Haagmans B. L. Osterhaus A. D. Smits S. L. **(**2013**).** Identification of multiple novel viruses, including a parvovirus and a hepevirus, in feces of red foxes. J Virol 87, 7758–7764 10.1128/JVI.00568-13 23616657 PMC3700315

[r9] Brookes S. M. Parsons G. Johnson N. McElhinney L. M. Fooks A. R. **(**2005a**).** Rabies human diploid cell vaccine elicits cross-neutralising and cross-protecting immune responses against European and Australian bat lyssaviruses. Vaccine 23, 4101–4109 10.1016/j.vaccine.2005.03.037 15964478

[r10] Brookes S. M. Aegerter J. N. Smith G. C. Healy D. M. Jolliffe T. A. Swift S. M. Mackie I. J. Pritchard J. S. Racey P. A. **& other authors (**2005b**).** European bat lyssavirus in Scottish bats. Emerg Infect Dis 11, 572–578 10.3201/eid1104.040920 15829196 PMC3320325

[r11] Brookes S. M. Healy D. M. Fooks A. R. **(**2006**).** Ability of rabies vaccine strains to elicit cross-neutralising antibodies. Dev Biol (Basel) 125, 185–193 16878476

[r12] Cleaveland S. Dye C. **(**1995**).** Maintenance of a microparasite infecting several host species: rabies in the Serengeti. Parasitology 111 (Suppl), S33–S47 10.1017/S0031182000075806 8632923

[r13] Cliquet F. Aubert M. Sagné L. **(**1998**).** Development of a fluorescent antibody virus neutralisation test (FAVN test) for the quantitation of rabies-neutralising antibody. J Immunol Methods 212, 79–87 10.1016/S0022-1759(97)00212-3 9671155

[r14] Dean D. J. Abelseth M. K. Atanasiu P. **(**1996**).** The fluorescent antibody test. In Laboratory Techniques in Rabies, pp. 88–93 Edited by Meslin F. X. Kaplan M. M. Koprowski H. Geneva: World Health Organisation

[r15] Dietzgen R. G. Calisher C. H. Kurath G. Kuzmin I. V. Rodriguez L. L. Stone D. M. Tesh R. B. Tordo N. Walker P. J. **& other authors (**2011**).** Rhabdoviridae. In Virus Taxonomy: Ninth Report of the International Committee on Taxonomy of Viruses. Edited by Andrew M. J. A. King M. Q. Carstens E. B. Lefkowitz E. J. Oxford: Elsevier

[r16] Estes R. D. **(**1992**).** Chapter 19: Family Viverridae. In The Behavior Guide to African Mammals, 1st edn California: University of California Press

[r17] Evans J. S. Horton D. L. Easton A. J. Fooks A. R. Banyard A. C. **(**2012**).** Rabies virus vaccines: is there a need for a pan-lyssavirus vaccine? Vaccine 30, 7447–7454 10.1016/j.vaccine.2012.10.015 23084854

[r18] Fooks A. **(**2004**).** The challenge of new and emerging lyssaviruses. Expert Rev Vaccines 3, 333–336 10.1586/14760584.3.4.333 15270628

[r19] Ge X. Li Y. Yang X. Zhang H. Zhou P. Zhang Y. Shi Z. **(**2012**).** Metagenomic analysis of viruses from bat fecal samples reveals many novel viruses in insectivorous bats in China. J Virol 86, 4620–4630 10.1128/JVI.06671-11 22345464 PMC3318625

[r20] Hanlon C. A. Kuzmin I. V. Blanton J. D. Weldon W. C. Manangan J. S. Rupprecht C. E. **(**2005**).** Efficacy of rabies biologics against new lyssaviruses from Eurasia. Virus Res 111, 44–54 10.1016/j.virusres.2005.03.009 15896401

[r21] Harris S. L. Aegerter J. N. Brookes S. M. McElhinney L. M. Jones G. Smith G. C. Fooks A. R. **(**2009**).** Targeted surveillance for European bat lyssaviruses in English bats (2003–06). J Wildl Dis 45, 1030–1041, 1041 10.7589/0090-3558-45.4.1030 19901379

[r22] Hayman D. T. Banyard A. C. Wakeley P. R. Harkess G. Marston D. Wood J. L. Cunningham A. A. Fooks A. R. **(**2011**).** A universal real-time assay for the detection of Lyssaviruses. J Virol Methods 177, 87–93 10.1016/j.jviromet.2011.07.002 21777619 PMC3191275

[r23] Hayman D. T. Fooks A. R. Rowcliffe J. M. McCrea R. Restif O. Baker K. S. Horton D. L. Suu-Ire R. Cunningham A. A. Wood J. L. N. **(**2012**).** Endemic Lagos bat virus infection in *Eidolon helvum*. Epidemiol Infect 140, 2163–2171 10.1017/S0950268812000167 22370126 PMC9152339

[r24] Healy D. M. Brookes S. M. Banyard A. C. Núñez A. Cosby S. L. Fooks A. R. **(**2013**).** Pathobiology of rabies virus and the European bat lyssaviruses in experimentally infected mice. Virus Res 172, 46–53 10.1016/j.virusres.2012.12.011 23274107

[r25] Hicks D. J. Nuñez A. Healy D. M. Brookes S. M. Johnson N. Fooks A. R. **(**2009**).** Comparative pathological study of the murine brain after experimental infection with classical rabies virus and European bat lyssaviruses. J Comp Pathol 140, 113–126 10.1016/j.jcpa.2008.09.001 19111840

[r26] Horton D. L. McElhinney L. M. Marston D. A. Wood J. L. Russell C. A. Lewis N. Kuzmin I. V. Fouchier R. A. Osterhaus A. D. **& other authors (**2010**).** Quantifying antigenic relationships among the lyssaviruses. J Virol 84, 11841–11848 10.1128/JVI.01153-10 20826698 PMC2977894

[r27] IUCN **(**2013**).** IUCN Red List of Threatened Species. Version 2013.1.

[r28] Johnson N. Vos A. Freuling C. Tordo N. Fooks A. R. Müller T. **(**2010**).** Human rabies due to lyssavirus infection of bat origin. Vet Microbiol 142, 151–159 10.1016/j.vetmic.2010.02.001 20188498

[r29] Kgaladi J. Nel L. H. Markotter W. **(**2013**).** Comparison of pathogenic domains of rabies and African rabies-related lyssaviruses and pathogenicity observed in mice. Onderstepoort J Vet Res 80, 511 10.4102/ojvr.v80i1.511 23718883

[r30] Koraka P. Martina B. E. Roose J. M. van Thiel P. P. van Amerongen G. Kuiken T. Osterhaus A. D. **(**2012**).** In vitro and in vivo isolation and characterization of Duvenhage virus. PLoS Pathog 8, e1002682 10.1371/journal.ppat.1002682 22654660 PMC3359985

[r31] Kuzmin I. V. Hughes G. J. Botvinkin A. D. Orciari L. A. Rupprecht C. E. **(**2005**).** Phylogenetic relationships of Irkut and West Caucasian bat viruses within the *Lyssavirus* genus and suggested quantitative criteria based on the N gene sequence for lyssavirus genotype definition. Virus Res 111, 28–43 10.1016/j.virusres.2005.03.008 15896400

[r32] Kuzmin I. V. Niezgoda M. Franka R. Agwanda B. Markotter W. Beagley J. C. Urazova O. Y. Breiman R. F. Rupprecht C. E. **(**2008a**).** Possible emergence of West Caucasian bat virus in Africa. Emerg Infect Dis 14, 1887–1889 10.3201/eid1412.080750 19046512 PMC2634633

[r33] Kuzmin I. V. Niezgoda M. Franka R. Agwanda B. Markotter W. Beagley J. C. Urazova O. Y. Breiman R. F. Rupprecht C. E. **(**2008b**).** Lagos bat virus in Kenya. J Clin Microbiol 46, 1451–1461 10.1128/JCM.00016-08 18305130 PMC2292963

[r34] Kuzmin I. V. Mayer A. E. Niezgoda M. Markotter W. Agwanda B. Breiman R. F. Rupprecht C. E. **(**2010**).** Shimoni bat virus, a new representative of the *Lyssavirus* genus. Virus Res 149, 197–210 10.1016/j.virusres.2010.01.018 20138934

[r35] Lembo T. Hampson K. Haydon D. T. Craft M. Dobson A. Dushoff J. Ernest E. Hoare R. Kaare M. **& other authors (**2008**).** Exploring reservoir dynamics: a case study of rabies in the Serengeti ecosystem. J Appl Ecol 45, 1246–1257 10.1111/j.1365-2664.2008.01468.x 22427710 PMC3303133

[r36] Li L. Shan T. Wang C. Côté C. Kolman J. Onions D. Gulland F. M. Delwart E. **(**2011**).** The fecal viral flora of California sea lions. J Virol 85, 9909–9917 10.1128/JVI.05026-11 21795334 PMC3196430

[r37] Lipkin W. I. Firth C. **(**2013**).** Viral surveillance and discovery. Curr Opin Virol 3, 199–204 10.1016/j.coviro.2013.03.010 23602435 PMC4310698

[r38] Lodmell D. L. Smith J. S. Esposito J. J. Ewalt L. C. **(**1995**).** Cross-protection of mice against a global spectrum of rabies virus variants. J Virol 69, 4957–4962 7609065 10.1128/jvi.69.8.4957-4962.1995 PMC189311

[r39] Malerczyk C. Selhorst T. Tordo N. Moore S. Müller T. **(**2009**).** Antibodies induced by vaccination with purified chick embryo cell culture vaccine (PCECV) cross-neutralize non-classical bat lyssavirus strains. Vaccine 27, 5320–5325 10.1016/j.vaccine.2009.06.095 19615958

[r40] Markotter W. Van Eeden C. Kuzmin I. V. Rupprecht C. E. Paweska J. T. Swanepoel R. Fooks A. R. Sabeta C. T. Cliquet F. Nel L. H. **(**2008**).** Epidemiology and pathogenicity of African bat lyssaviruses. Dev Biol (Basel) 131, 317–325 18634494

[r41] Marston D. A. Horton D. L. Ngeleja C. Hampson K. McElhinney L. M. Banyard A. C. Haydon D. Cleaveland S. Rupprecht C. E. **& other authors (**2012a**).** Ikoma lyssavirus, highly divergent novel lyssavirus in an African civet. Emerg Infect Dis 18, 664–667 10.3201/eid1804.111553 22469151 PMC3309678

[r42] Marston D. A. Ellis R. J. Horton D. L. Kuzmin I. V. Wise E. L. McElhinney L. M. Banyard A. C. Ngeleja C. Keyyu J. **& other authors (**2012b**).** Complete genome sequence of Ikoma lyssavirus. J Virol 86, 10242–10243 10.1128/JVI.01628-12 22923801 PMC3446578

[r43] Phan T. G. Kapusinszky B. Wang C. Rose R. K. Lipton H. L. Delwart E. L. **(**2011**).** The fecal viral flora of wild rodents. PLoS Pathog 7, e1002218 10.1371/journal.ppat.1002218 21909269 PMC3164639

[r44] Robardet E. Demerson J. M. Andrieu S. Cliquet F. **(**2012**).** First European interlaboratory comparison of tetracycline and age determination with red fox teeth following oral rabies vaccination programs. J Wildl Dis 48, 858–868 10.7589/2011-07-205 23060487

[r45] Sabeta C. T. Shumba W. Mohale D. K. Miyen J. M. Wandeler A. I. Nel L. H. **(**2008**).** Mongoose rabies and the African civet in Zimbabwe. Vet Rec 163, 580 10.1136/vr.163.19.580 18997193

[r46] Turmelle A. S. Allen L. C. Jackson F. R. Kunz T. H. Rupprecht C. E. McCracken G. F. **(**2009**).** Ecology of rabies virus exposure in colonies of Brazilian free-tailed bats (*Tadarida brasiliensis*) at natural and man-made roosts in Texas. Vector Borne Zoonotic Dis 10, 165–175 19492942 10.1089/vbz.2008.0163 PMC2944840

[r47] van den Brand J. M. van Leeuwen M. Schapendonk C. M. Simon J. H. Haagmans B. L. Osterhaus A. D. Smits S. L. **(**2012**).** Metagenomic analysis of the viral flora of pine marten and European badger feces. J Virol 86, 2360–2365 10.1128/JVI.06373-11 22171250 PMC3302375

[r48] Wilbur L. A. Aubert M. **(**1996**).** NIH test for potency. In Laboratory Techniques in Rabies, 4th edn, pp. 360–368 Edited by Meslin F. X. Kaplan M. M. Koprowski H. Geneva: World Health Organisation

[r49] Wright E. McNabb S. Goddard T. Horton D. L. Lembo T. Nel L. H. Weiss R. A. Cleaveland S. Fooks A. R. **(**2009**).** A robust lentiviral pseudotype neutralisation assay for in-field serosurveillance of rabies and lyssaviruses in Africa. Vaccine 27, 7178–7186 10.1016/j.vaccine.2009.09.024 19925950 PMC2789314

